# Perprocedural Heparinization in Non-cardiac Arterial Procedures: The Current Practice in the Netherlands

**DOI:** 10.1177/15266028231199714

**Published:** 2023-09-25

**Authors:** Liliane C. Roosendaal, Max Hoebink, Arno M. Wiersema, Kak K. Yeung, Jan D. Blankensteijn, Vincent Jongkind

**Affiliations:** 1 Department of Vascular Surgery, Dijklander Ziekenhuis, Hoorn, The Netherlands; 2 Department of Vascular Surgery, Amsterdam UMC, location VUmc, Amsterdam, The Netherlands; 3 Microcirculation, Amsterdam Cardiovascular Sciences, Amsterdam, The Netherlands

**Keywords:** heparin, non-cardiac arterial procedures, vascular surgical procedures, activated clotting time

## Abstract

**Purpose:**

Heparin is the most widely-used anticoagulant to prevent thrombo-embolic complications during non-cardiac arterial procedures (NCAP). Unfortunately, there is a lack of evidence and consequently non-uniformity in guidelines on perprocedural heparin management. Detailed insight into the current practice of antithrombotic strategies during NCAP in the Netherlands is important, aiming to identify potential optimal protocols and local differences concerning perprocedural heparinization.

**Materials and Methods:**

A comprehensive online survey was distributed electronically to vascular surgeons of every hospital in the Netherlands in which NCAP were performed. Data were collected from September 2020 to October 2021.

**Results:**

The response rate was 90% (53/59 hospitals). During NCAP, all surgeons generally administered heparin before arterial clamping. In 74% (39/54) of hospitals, a single heparin dosing protocol was used for all types of patients and vascular procedures. In 40%, there was no uniformity in heparin dosing between vascular surgeons. Depending on the procedure, a fixed bolus heparin, predominantly 5000 IU, was administered in 73% to 93%. In the remaining hospitals (7%–27%), a bodyweight-based heparin protocol was used, with an initial dose of 70 or 100 IU/kg. A minority (28%) monitored the effect of heparin in patients using the activated clotting time add (ACT) after activated clotting time. Target values varied between 180 and 250 seconds or 2 times the baseline ACT.

**Conclusion:**

This survey demonstrates considerable variability in perprocedural heparinization during NCAP in the Netherlands. Future research on heparin dosing is needed to harmonize and optimize heparin dosage protocols and contemporary guidelines during NCAP, and thereby improve vascular surgical care and patient safety.

**Clinical Impact:**

This survey demonstrated persisting intra- and inter-hospital variability in perprocedural heparinization during non-cardiac arterial procedures (NCAP) in the Netherlands. The observed variability in heparinization strategies highlights the need for high quality evidence on perprocedural anticoagulation strategies. This is needed in order to harmonize and optimize heparin dosage protocols and contemporary guidelines and thereby improve vascular surgical patient care. Based on the current results, an international survey will be conducted by the authors to gain additional insight into the antithrombotic strategies used during NCAP, aiming to harmonize anticoagulation protocols worldwide.

## Introduction

Throughout the years, outcomes of non-cardiac arterial procedures (NCAP) have improved significantly.^1,2^ Yet, perprocedural and postoperative arterial thrombo-embolic complications (TEC) are still common, with incidences between 4.3% and 12%.^3,4^ Optimal anticoagulation is important for safe and effective NCAP. Adequate dosing of heparin is thought to benefit patients to lower the risk of bleeding complications and TEC.^4–6^ However, heparin has a nonlinear dose response curve and elimination curve, which causes an unpredictable effect in the individual patient.^7^ To ensure sufficient anticoagulation in every patient throughout the procedure, the effect of heparin could be measured using anticoagulation tests. Thrombin time, activated partial thromboplastin time (aPTT), and anti-Xa are anticoagulation tests that have been used. These tests have limitations in accurately measuring the effect of heparin during NCAP or require a significant amount of time to obtain results. Therefore, activated clotting time (ACT) is the most suitable coagulation test which can be used to monitor the effect of perprocedural heparinization. In anticipation of the first randomised controlled trial on monitoring anticoagulation during NCAP, level 1 evidence is still lacking.^8–10^ In addition, recommendations in clinical practice guidelines on ACT monitoring and heparin reversal using protamine during NCAP are limited. If recommendations are made, they vary widely between guidelines and are based on non-randomised, primarily retrospective studies or on no literature at all.^11–18^ However, a shift toward personalized medicine could involve tailoring anticoagulation therapy to each patient’s specific needs, potentially improving treatment outcomes and reducing the risk of adverse events, which could potentially reduce health care costs associated with complications and hospital readmissions. Recent research suggests, for example, that weight-based dosing leads to more adequate anticoagulation during NCAP, as well as dose adjustments based on sex.^4,19^

A previous survey study on perprocedural thromboprophylaxis management in the Netherlands in 2011 showed 5000 IU of heparin, regardless of body weight, to be the predominant dosage during vascular procedures. The vast majority (89%) of vascular surgeons reported to dose heparin without ACT- or aPTT measurements.^20^ Because of the lack of evidence, and consequently non-uniformity in available guidelines, local differences concerning perprocedural heparinization and monitoring its effect in the Netherlands might still be present. In addition, an overview of the current practice in perprocedural heparinization strategies might aid in identifying variability, showing potential knowledge gaps or might help to reach consensus on optimal heparinization for the individual patient. Therefore, a new survey was conducted to provide insight into the current practice of perprocedural heparinization during NCAP in the Netherlands.

## Materials and Methods

A comprehensive online survey was designed to examine the perprocedural heparinization strategies during NCAP. The survey was sent to vascular surgeons of every hospital in the Netherlands where NCAP were performed. Vascular surgeons responded to the survey, after consultation with the vascular surgical team to identify concurrent practice, and using their local protocols.

The survey was sent in September 2020 using the cloud-based Electronic Data Capture platform “Castor EDC.”^21^ Reminder emails were sent to the vascular surgeons if the survey was not answered. When no reply was received after 3 reminders and direct contact, the survey was sent to another vascular surgeon of the same hospital. The last reminder was sent in October 2021. Communications contained a case-specific single-click link to the survey. No financial compensation was offered to participants.

The survey covered questions about perprocedural heparinization, monitoring, and reversal of heparin. Questions were asked about heparin dosage protocols during different surgical procedures: carotid endarterectomy (CEA), carotid artery stenting (CAS), endovascular aneurysm repair (EVAR), thoracic endovascular aneurysm repair (TEVAR), open abdominal aortic aneurysm repair (OAAA), fenestrated/branched endovascular aneurysm repair (FEVAR/BEVAR), femoral endarterectomy, femoral-distal bypass surgery, and peripheral percutaneous transluminal angioplasty (PTA).

The survey contained 7 general questions and 16 questions per heparin protocol used. Protocol-specific questions comprised the starting dose of heparin used, specifications about the use of perprocedural ACT measurements, and the potential use of protamine.

### Statistical Analysis

Descriptive analyses were performed using Statistical Package for the Social Sciences (SPSS) version 28 (Armonk, New York: IBM Corp.).

## Results

### Response

Vascular surgeons from eight academic hospitals and 51 peripheral hospitals were invited to complete the survey. In total, 53 out of 59 (90%) vascular surgeons from different hospitals completed the survey. Seven respondents worked in an academic hospital and 46 in a peripheral hospital.

### Vascular Surgery Procedures

In the participating hospitals, the following procedures were performed at time of the survey: CEA: 45 hospitals (85%); CAS: 18 hospitals (34%); EVAR: 49 hospitals (92%); TEVAR: 21 hospitals (40%); OAAA: 50 hospitals (94%); FEVAR and/or BEVAR: 24 hospitals (45%). Femoral endarterectomy, femoral-distal bypass surgery, and PTA were performed in all participating hospitals (Table 1).

**Table 1.** Width of the Vascular Surgical Range of Procedures Being Performed.

Procedure	Number of hospitals where procedures were performed
CEA	45 (85%)
CAS	18 (34%)
EVAR	49 (92%)
TEVAR	21 (40%)
OAAA	50 (94%)
FEVAR/BEVAR	24 (45%)
Femoral endarterectomy	53 (100%)
Femoral-distal bypass surgery	53 (100%)
Peripheral PTA	53 (100%)

Abbreviations: CAS, carotid artery stenting; CEA, carotid endarterectomy; EVAR, endovascular aneurysm repair; FEVAR/BEVAR, fenestrated/branched endovascular aneurysm repair; OAAA, open abdominal aortic aneurysm repair; PTA, percutaneous transluminal angioplasty; TEVAR, thoracic endovascular aneurysm repair.

### Heparin Dosing Protocols

In all hospitals, heparin was administered during elective NCAP. Specific individual cases were mentioned where less or no heparin at all was administered: if a direct oral anticoagulant was continued, during an arteriovenous-fistula procedure, and if dual antiplatelet therapy was used and not discontinued. One hospital reported only local intra-arterial heparin administration in both lower extremities during open AAA surgery.

In 39 hospitals (74%), a single standard heparin dosing protocol was used for all types of vascular procedures. In 9 hospitals (17%), more than 1 heparin dosing protocol was used, whereas in 5 hospitals (9%), there was no standard dosing protocol. Thirty-two (60%) responded the protocol was used in the same way by all staff members. A single heparin dosing protocol was used by >75% of staff in 17 (32%) hospitals. In 4 hospitals (7.5%), 50% to 75% of the staff members used the same protocol. In 26 of the cases (49%), the same heparin protocol was used by the interventional radiologists of the responding hospital.

Detailed information on heparin dosing protocols per procedure can be found in Table 2.

**Table 2.** Heparin Dosing Protocols per Type of Procedure.

		Starting dose				Follow-up dose			
		Fixed dose		Based on bodyweight		No follow-up dose	Based on ACT	Potentially, based on perprocedural coagulation/length surgery	Based on protocol
Procedures	Number of heparin protocol received	5000 IU No. (%)	Other No. (%)	70 IU/kg No. (%)	100 IU/kg No. (%)	No. (%)	No. (%)	No. (%)	No. (%)
CEA	42	33 (78)	3 (7)	2 (5)	4 (10)	4 (10)	8 (19)	27 (64)	3 (7)
CAS	5	4 (80)	—	1 (20)	—	1 (20)	1 (20)	2 (40)	1 (20)
EVAR	44	33 (75)	4 (9)	3 (7)	4 (9)	4 (9)	8 (18)	30 (68)	2 (5)
TEVAR	19	12 (62)	2 (11)	3 (16)	2 (11)	2 (11)	6 (32)	9 (46)	2 (11)
FEVAR/BEVAR	20	13 (65)	2 (10)	2 (10)	3 (15)	2 (10)	8 (40)	9 (45)	1 (5)
OAAA	47	35 (75)	5 (11)	3 (6)	4 (9)	6 (13)	8 (17)	31 (66)	2 (4)
Femoral endarterectomy	46	36 (78)	3 (7)	3 (7)	4 (8)	6 (14)	7 (15)	31 (67)	2 (4)
Femoral-distal bypass surgery	47	37 (79)	3 (6)	2 (4)	5 (11)	6 (13)	8 (17)	30 (64)	3 (6)
Peripheral PTA	41	36 (88)	2 (5)	2 (5)	1 (2)	4 (10)	6 (15)	29 (70)	2 (5)

Other fixed heparin dose = other fixed starting dose than 5000 IU.

Abbreviations: ACT, activated clotting time; CAS, carotid artery stenting; CEA, carotid endarterectomy; EVAR, endovascular aneurysm repair; FEVAR/BEVAR, fenestrated/branched endovascular aneurysm repair; OAAA, open abdominal aortic aneurysm repair; PTA, percutaneous transluminal angioplasty; TEVAR, thoracic endovascular aneurysm repair.

### Administration of Heparin

Depending on the procedure performed, a fixed dose of heparin was administered in 73% to 93% of the hospitals, primarily as a bolus of 5000 IU (62%–88%) (Table 2). In the remaining hospitals (7%–27%), a bodyweight-depended heparin protocol was used, with an initial heparin dose of 70 or 100 IU/kg. Follow-up doses of heparin were administered in 9% to 20% of hospitals. The predominant reason for additional heparin administration was “based on perprocedural coagulation status or length of surgery” (40%–70%). Other reasons for follow-up doses were “based on ACT” (15%–40%) and “based on protocol, additional 2500 IU” (5%–20%).

### Monitoring the Effect of Heparin

Fifteen hospitals (28%) reported the use of perprocedural heparinization monitoring during one or multiple NCAP: ACT (n=14) or aPTT (n=1) measurements.

Target ACTs varied between 180 and 250 seconds or were 2 times the baseline ACT. First ACT after heparinization was measured after 5 minutes (3–30 minutes). Second ACT followed after 30 minutes (30–90 minutes), and follow-up ACT measurements every 30 minutes (30–90 minutes) until the end of the procedures. Target ACTs at closure were provided by 8 hospitals, depending on the procedure. The target ACTs at closure varied between 150 and 250 seconds. Detailed information per procedure can be found in Table 3.

**Table 3.** ACT Target Values and Moment of ACT Measurements per Type of Procedure.

Procedures	Target ACT, s	Target ACT, No. (%)	First ACT after heparin, min (%)	Second ACT after heparin, min (%)	Follow-up ACT, min (%)	Target ACT at closure, s	Target ACT at closure, No. (%)
CEA	180 200 200-220 200-250 2 times baseline ACT	1 (13) 3 (50) 1 (13) 1 (13) 1 (13)	5 (43) 10 (14) 15 (29) 20 (14)	30 (57) 45 (13) 60 (13) Missing (13)	Every 30 (57) Every 60 (13) Depending on clamping time (13) Missing (13)	180 150	4 (57) 3 (43)
CAS	180	1 (100)	15 (100)	30 (100)	Every 30 (100)	150	1 (100)
EVAR	180 200 200-220 2 times baseline ACT 200-250 or 2 times baseline ACT	1 (11) 4 (44) 1 (11) 2 (22) 1 (11)	5 (33) 10 (11) 15 (22) 20 (11) 30 (11) Missing (11)	30 (44) 45 (11) 60 (11) 90 (11) Missing (11)	Every 30 (44) Every 60 (11) Depending on clamping time (11) Missing (33)	150 180 170	2 (29) 4 (57) 1 (14)
TEVAR	180 200 2 times baseline ACT	1 (14) 4 (57) 2 (29)	5 (29) 10 (14) 15 (29) 20 (14) 30 (14)	30 (43) 45 (14) 60 (14) 90 (14) Missing (14)	Every 30 (43) Every 60 (14) Depending on clamping time (14) Missing (29)	150 170 180	2 (40) 1 (20) 2 (40)
FEVAR/BEVAR	200 200-250 2 times baseline ACT 200-250 or 2 times baseline ACT	6 (55) 2 (18) 2 (18) 1 (9)	3 (9) 4 (9) 5 (18) 10 (9) 15 (27) 20 (9) 30 (18)	30 (27) 45 (9) 60 (18) Missing (45)	Every 30 (27) Every 60 (18) Depending on clamping time (9) Missing (45)	150 170 180 250	4 (50) 1 (13) 2 (25) 1 (13)
OAAA	180 200 200-220 200-250 2 times baseline ACT	1 (11) 4 (44) 1 (11) 1 (11) 2 (22)	5 (33) 10 (11) 15 (22) 20 (11) 30 (11) Missing (11)	30 (44) 45 (11) 60 (11) 90 (11) Missing (22)	Every 30 (44) Every 60 (11) Depending on clamping time (11) Missing (33)	150 170 180	2 (29) 1 (14) 4 (57)
Femoral endarterectomy	180 200 200-220 2 times baseline ACT 200-250 or 2 times baseline ACT	1 (14) 3 (43) 1 (14) 1 (14) 1 (14)	5 (43) 10 (14) 15 (29) 20 (14)	30 (57) 45 (14) 60 (14) Missing (14)	Every 30 (57) Every 60 (14) Depending on clamping time (14) Missing (14)	150 170 180	2 (33) 1 (17) 3 (50)
Femoral-distal bypass surgery	180 200 200-220 2 times baseline ACT 200-250 or 2 times baseline ACT	1 (13) 3 (38) 1 (13) 2 (25) 1 (13)	5 (50) 10 (13) 15 (25) 20 (13)	30 (50) 45 (25) 60 (13) Missing (13)	Every 30 (50) Every 60 (13) Every 90 (13) Depending on clamping time (13) Missing (13)	150 170 180	3 (43) 1 (14) 3 (43)
Peripheral PTA	180 200 200-220 2 times baseline ACT 200-250 or 2 times baseline ACT	1 (17) 1 (17) 1 (17) 2 (32) 1 (17)	5 (50) 10 (17) 15 (17) 20 (17)	30 (50) 45 (32) 60 (17)	Every 30 (50) Every 60 (17) Every 90 (17) Missing (17)	150 170 180	3 (50) 1 (17) 2 (33)

Abbreviations: ACT, activated clotting time; CAS, carotid artery stenting; CEA, carotid endarterectomy; EVAR, endovascular aneurysm repair; FEVAR/BEVAR, fenestrated/branched endovascular aneurysm repair; OAAA, open abdominal aortic aneurysm repair; PTA, percutaneous transluminal angioplasty; TEVAR, thoracic endovascular aneurysm repair.

Various ACT measurement devices were used; the Hemochron Signature Elite was predominantly used. Specifications of the ACT measurement devices used are displayed in Supplemental Table 1.

### Administration of Protamine

Depending on the procedure, protamine was rarely or never used according to 42% to 80% of hospitals. In the hospitals using ACT-guided heparinization, protamine was usually administered based on ACT values. Other hospitals using protamine based their protamine dosage on the administered heparin dose. Detailed information on the administration of protamine per procedure can be found in Table 4.

**Table 4.** Protamine Dosing Protocols per Type of Procedure.

Procedures	Protamine protocol, No	Rarely/never, No. (%)	Fixed dose independent of ACT, No. (%)	Based on heparin dose, No. (%)	Based on ACT, No. (%)
CEA	42	22 (52)	3 (7)	13 (31)	4 (10)
CAS	5	4 (80)	—	—	1 (20)
EVAR	44	27 (61)	4 (9)	9 (21)	4 (9)
TEVAR	19	8 (42)	2 (11)	6 (31)	3 (16)
FEVAR/BEVAR	20	10 (50)	3 (15)	4 (20)	3 (15)
OAAA	47	28 (59)	4 (9)	11 (23)	4 (9)
Femoral endarterectomy	46	29 (63)	4 (9)	10 (22)	3 (6)
Femoral-distal bypass surgery	47	27 (57)	3 (6)	13 (28)	4 (9)
Peripheral PTA	40	25 (63)	4 (10)	7 (17)	4 (10)

Abbreviations: ACT, activated clotting time; CAS, carotid artery stenting; CEA, carotid endarterectomy; EVAR, endovascular aneurysm repair; FEVAR/BEVAR, fenestrated/branched endovascular aneurysm repair; OAAA, open abdominal aortic aneurysm repair; PTA, percutaneous transluminal angioplasty; TEVAR, thoracic endovascular aneurysm repair.

### Evaluation of Satisfaction With Current Protocol

In 12 hospitals (23%), vascular surgeons were not satisfied with the current dosing protocol on account of the heparin dosing protocol not being adjusted to individual patient characteristics, such as weight and gender. In addition, some of the hospitals (n=3) indicated they would like to measure the effect of heparin, but no ACT measuring device was available.

## Discussion

For this comprehensive survey, a high response rate was achieved (90%), and therefore, a complete overview of heparinization strategies used during CEA, CAS, EVAR, TEVAR, OAAA, FEVAR/BEVAR, femoral endarterectomy, femoral-distal bypass surgery, and PTA in the Netherlands could be constructed. High variety in heparinization protocols was identified among hospitals performing NCAP, but also within each hospital, as only 60% of staff members adhered to the same heparinization protocol. The latter is important because standardization of procedures is crucial to maintain consistent results, save time, and lead to better outcomes.^22,23^ Therefore, it is strongly recommended to use one optimal anticoagulant protocol, to provide the safest treatment for each patient. Unfortunately, it is still unclear what the most optimal anticoagulation protocol is during NCAP.

A survey conducted in 2011 among vascular surgeons from the Netherlands led to the initiation of research over the past decade aimed at exploring the optimal dosage of heparin for NCAP.^20^ Findings indicated that a standardized bolus of 5000 IU of heparin does not provide sufficient and safe heparinization.^6^ However, when examining the heparinization protocols currently used in participating hospitals, an initial heparin dose of 5000 IU was still predominantly administered (62%–88%).

Prior research has showed that weight-based heparin dosing results in adequate anticoagulation in most patients.^4^ This was used by a minority of the respondents. The current survey indicates that when weighed-based dosing was used, an initial heparin dose ranging from 70 to 100 IU/kg was used, which is consistent with recent NCAP guidelines.^12,24^ Weight-based dosing provides the advantage of a more personalized and tailored approach to heparin administration, taking into account patient’s specific characteristics.^4^ This is of significant importance, as indicated by the fact that 23% of vascular surgeons expressed dissatisfaction with their current dosing protocol on account of their heparin dosing protocol not being adjusted to individual patient characteristics.

Besides to tailoring the heparinization protocol based on patient characteristics, adapting the protocol to the specific type of NCAP being performed could potentially lead to improved outcomes. Although evidence is currently lacking, it is conceivable that, for instance, extensive lengthy open procedures may necessitate a different anticoagulation strategy compared with a shorter endovascular procedure.^25^ Furthermore, due to the short half-life of heparin, timely procedures may require additional heparin administration to maintain adequate anticoagulation.^26^ A recent study described inadequate anticoagulation 30 minutes after the initial heparin administration for the majority of patients.^19^ In the current survey, additional heparin doses were administered in 80% to 91% of hospitals, predominantly based on the length of the procedure or on perceived coagulation during the procedure.

Due to the pharmacokinetics of heparin, the anticoagulant effect is unpredictable in the individual patient. To address this variability, monitoring the effect of heparin using the ACT can be performed, which enables further personalization of the heparinization protocol. In a minority (28%) of hospitals, the effect of heparin was monitored using the ACT during at least one of the described NCAP. The time point of the first ACT measurement varied widely between centers (5–30 minutes). This is surprising, as measuring the first ACT after 30 minutes appears inappropriate considering that the peak anticoagulation response typically occurs within 1 minute after administration.^27^ Target ACT value was 200 seconds in most hospitals and 1 hospital mentioned a target ACT of 180 seconds. However, optimal target ACT is not known for NCAP. Several previous studies suggest a target ACT between 200 and 250 seconds, but large studies on this subject have not yet been performed and the target ACT may differ depending on the procedure performed.^4,28^ In addition, ACT values are known to differ depending on the measurement device and cartridges used, with low correlation between different devices.^29,30^

Protamine was rarely administered. According to the most recent guidelines for carotid procedures of the European Society for Vascular Surgery (ESVS), reversal of heparin using protamine should be considered to prevent neck hematomas requiring re-exploration.^13,31^ Protamine was rarely or never considered in roughly 50% of hospitals in both the current study and in a survey in the United Kingdom.^32^ During all other NCAP, protamine was administered even less frequently. No information was collected on the reason why protamine is rarely administered, but a potential reason could be the risk of an immediate adverse reaction to protamine. From literature this risk is 10.7%, so there might be room for improvement.^33^

Previous international surveys on the use of antithrombotic medication during vascular surgery and interventional radiology showed the use of heparin in 90% to 100% of cases during NCAP.^20,34–36^ Typically, a bolus of 5000 IU is administered, with calculated individual heparin dosages less often. In addition, the ACT was used to monitor the effect of heparin by 43% of ESVS surgeons, and in 80% of SVS surgeons during CEA. This differs strongly from our findings from the Netherlands (28% in the current survey, 11% in a survey from 2011). Unfortunately, in the abovementioned surveys, no information was provided regarding additional heparin doses, target ACTs, and heparin measurement devices used. In addition, lower response rates were found compared with the current survey. Furthermore, one survey was voluntary, which could lead to bias because several vascular surgeons from the same hospital, or mainly vascular surgeons with a specific interest in anticoagulation, might have completed the survey.^37^ In contrast, the current survey was completed by nearly all peripheral and academic NCAP centers in the Netherlands, making the outcomes representative of the current practice of heparinization during NCAP in the Netherlands.

To keep the survey concise to reach this high response rate, it was solely focused on acquiring data regarding perprocedural anticoagulation, without including data on postprocedural anticoagulation strategies. Another limitation of the current survey is the lack of information regarding the number of NCAP conducted and the size of the participating hospitals, as these data were not included in the survey.

## Conclusion

Compared with a similar survey in 2011, this survey demonstrates persisting intra-hospital and inter-hospital variability in perprocedural heparinization during NCAP in the Netherlands. Future research on heparin dosing is needed to harmonize and optimize heparin dosage protocols and contemporary guidelines during NCAP and thereby improve vascular surgical care and patient safety. Since level 1 evidence on monitoring anticoagulation during NCAP is currently lacking, an international survey will be conducted by the authors to gain more insight into the current practice of anticoagulant use during NCAP, to harmonize anticoagulation protocols worldwide.

## Supplemental Material

sj-docx-1-jet-10.1177_15266028231199714 – Supplemental material for Perprocedural Heparinization in Non-cardiac Arterial Procedures: The Current Practice in the Netherlands Supplemental material, sj-docx-1-jet-10.1177_15266028231199714 for Perprocedural Heparinization in Non-cardiac Arterial Procedures: The Current Practice in the Netherlands by Liliane C. Roosendaal, Max Hoebink, Arno M. Wiersema, Kak K. Yeung, Jan D. Blankensteijn and Vincent Jongkind in Journal of Endovascular Therapy

## References

[1] Smilowitz NR Gupta N Ramakrishna H , et al. Perioperative major adverse cardiovascular and cerebrovascular events associated with non-cardiac surgery. JAMA Cardiol. 2017;2(2):181–187. doi:10.1001/JAMACARDIO.2016.4792. 28030663 PMC5563847

[2] Egorova NN Guillerme S Gelijns A , et al. An analysis of the outcomes of a decade of experience with lower extremity revascularization including limb salvage, lengths of stay, and safety. J Vasc Surg. 2010;51(4):878–885. doi:10.1016/J.JVS.2009.10.102. 20045618

[3] Geraedts ACM Alberga AJ Koelemay MJW , et al. Short-term outcomes of open surgical abdominal aortic aneurysm repair from the Dutch Surgical Aneurysm Audit. BJS Open. 2021;5(5):zrab086. doi:10.1093/BJSOPEN/ZRAB086. PMC8438252 34518868

[4] Doganer O Roosendaal LC Wiersema AM , et al. Weight based heparin dosage with activated clotting time monitoring leads to adequate and safe anticoagulation in non-cardiac arterial procedures. Ann Vasc Surg. 2022;84:327–335. doi:10.1016/J.AVSG.2022.01.029. 35248743

[5] Wiersema AM Jongkind V Bruijninckx CM , et al. Prophylactic perioperative anti-thrombotics in open and endovascular abdominal aortic aneurysm (AAA) surgery: a systematic review. Eur J Vasc Endovasc Surg. 2012;44(4):359–367. doi:10.1016/j.ejvs.2012.06.008. 22831869

[6] Doganer O Jongkind V Blankensteijn JD , et al. A standardized bolus of 5 000 IU of heparin does not lead to adequate heparinization during non-cardiac arterial procedures. Ann Vasc Surg. 2020;0(0):1–7. doi:10.1016/j.avsg.2020.07.035. 32768536

[7] de Swart CA Nijmeyer B Roelofs JM , et al. Kinetics of intravenously administered heparin in normal humans. Blood. 1982;60(6):1251–1258. doi:10.1182/BLOOD.V60.6.1251.1251. 7139119

[8] Nath FC Muller DW Rosenschein U , et al. Heparin monitoring during coronary intervention: activated clotting time versus activated partial thromboplastin time. Can J Cardiol. 1993;9(9):797–801. 8281479

[9] Chew DP Bhatt DL Lincoff AM , et al. Defining the optimal activated clotting time during percutaneous coronary intervention: aggregate results from 6 randomized, controlled trials. Circulation. 2001;103(7):961–966. doi:10.1161/01.CIR.103.7.961. 11181470

[10] Wiersema AM Roosendaal LC Koelemaij MJW , et al. ACTION-1: study protocol for a randomised controlled trial on ACT-guided heparinization during open abdominal aortic aneurysm repair. Trials. 2021;22(1):1–16. doi:10.1186/S13063-021-05552-7/FIGURES/3. 34538275 PMC8449992

[11] Chaikof EL Dalman RL Eskandari MK , et al. The Society for Vascular Surgery practice guidelines on the care of patients with an abdominal aortic aneurysm. J Vasc Surg. 2018;67(1):2–77e2. doi:10.1016/j.jvs.2017.10.044. 29268916

[12] Wanhainen A Verzini F Van Herzeele I , et al. European Society for Vascular Surgery (ESVS) 2019 clinical practice guidelines on the management of abdominal aorto-iliac artery aneurysms. Eur J Vasc Endovasc Surg. 2019;57(1):8–93. doi:10.1016/j.ejvs.2018.09.020. 30528142

[13] Naylor AR Ricco JB de Borst GJ , et al. Editor’s choice—management of atherosclerotic carotid and vertebral artery disease: 2017 clinical practice guidelines of the European Society for Vascular Surgery (ESVS). Eur J Vasc Endovasc Surg. 2018;55(1):3–81. doi:10.1016/J.EJVS.2017.06.021. 28851594

[14] AbuRahma AF Avgerinos ED Chang RW , et al. Society for Vascular Surgery clinical practice guidelines for management of extracranial cerebrovascular disease. J Vasc Surg. 2022;75(1S):4S–22S. doi:10.1016/J.JVS.2021.04.073. 34153348

[15] Conte MS Bradbury AW Kolh P , et al. Global vascular guidelines on the management of chronic limb-threatening ischemia. J Vasc Surg. 2019;69(6S):3S–125S.e40. doi:10.1016/J.JVS.2019.02.016. PMC8365864 31159978

[16] Upchurch GR Jr Escobar GA Azizzadeh A , et al. Society for vascular surgery clinical practice guidelines of thoracic endovascular aortic repair for descending thoracic aortic aneurysms. J Vasc Surg. 2021;73(1S):55S–83S. doi:10.1016/J.JVS.2020.05.076. 32628988

[17] Riambau V Böckler D Brunkwall J , et al. Editor’s choice—management of descending thoracic aorta diseases: clinical practice guidelines of the European Society for Vascular Surgery (ESVS). Eur J Vasc Endovasc Surg. 2017;53(1):4–52. doi:10.1016/J.EJVS.2016.06.005. 28081802

[18] Wiersema AM Jongkind V Bruijninckx CM , et al. Prophylactic perioperative anti-thrombotics in open and endovascular abdominal aortic aneurysm (AAA) surgery: a systematic review. Eur J Vasc Endovasc Surg. 2012;44(4):359–367. doi:10.1016/J.EJVS.2012.06.008. 22831869

[19] Roosendaal LC Wiersema AM Smit JW , et al. Sex differences in response to administration of heparin during non-cardiac arterial procedures. Eur J Vasc Endovasc Surg. 2022;64(5):557–565. doi:10.1016/J.EJVS.2022.08.005. 35973666

[20] Wiersema A Bruijninckx C Reijnen M , et al. Perioperative prophylactic antithrombotic strategies in vascular surgery: current practice in the Netherlands. J Cardiovasc Surg (Torino). 2015;56(1):119–125. 23337406

[21] *Castor Electronic Data Capture* ; 2019. https://www.castoredc.com/electronic-data-capture-system. Accessed August 28, 2023.

[22] Dalton JE Zidar DA Udeh BL , et al. Practice variation among hospitals in revascularization therapy and its association with procedure-related mortality. Med Care. 2016;54(6):623–631. doi:10.1097/MLR.0000000000000536. 27050445 PMC4865419

[23] Grimshaw JM Russell IT. Effect of clinical guidelines on medical practice: a systematic review of rigorous evaluations. Lancet (London, England). 1993;342(8883):1317–1322. doi:10.1016/0140-6736(93)92244-N. 7901634

[24] Rich K Treat-Jacobson D DeVeaux T , et al. Society for Vascular Nursing-Carotid Endarterectomy (CEA) updated nursing clinical practice guideline. J Vasc Nurs. 2017;35(2):90–111. doi:10.1016/J.JVN.2017.03.004. 28527733

[25] Patrone L Pasqui E Sheahan K , et al. Optimal activated clotting time during peripheral artery endovascular procedures: cutting the gordian knot. J Cardiovasc Surg (Torino). 2023;64(3):240–246. doi:10.23736/S0021-9509.23.12703-0. 37260152

[26] Estes JW. Clinical pharmacokinetics of heparin. Clin Pharmacokinet. 1980;5(3):204–220. doi:10.2165/00003088-198005030-00002. 6993082

[27] Heres EK Speight K Benckart D , et al. The clinical onset of heparin is rapid. Anesth Analg. 2001;92(6):1391–1395. doi:10.1097/00000539-200106000-00006. 11375810

[28] Kasapis C Gurm HS Chetcuti SJ , et al. Defining the optimal degree of heparin anticoagulation for peripheral vascular interventions insight from a large, regional, multicenter registry. Circ Cardiovasc Interv. 2010;3(6):593–601. doi:10.1161/CIRCINTERVENTIONS.110.957381. 21062999

[29] Chia S Van Cott EM Raffel OC , et al. Comparison of activated clotting times obtained using Hemochron and Medtronic analysers in patients receiving anti-thrombin therapy during cardiac catheterisation. Thromb Haemost. 2009;101(3):535–540. doi:10.1160/TH08-08-0528. 19277416

[30] Lee JM Park EY Kim KM , et al. Comparison of activated clotting times measured using the Hemochron Jr Signature and Medtronic ACT Plus during cardiopulmonary bypass with acute normovolemic haemodilution. J Int Med Res. 2018;46(2):873–882. doi:10.1177/0300060517731952. 28974132 PMC5971518

[31] Kakisis JD Antonopoulos CN Moulakakis KG , et al. Protamine reduces bleeding complications without increasing the risk of stroke after carotid endarterectomy: a meta-analysis. Eur J Vasc Endovasc Surg. 2016;52(3):296–307. doi:10.1016/J.EJVS.2016.05.033. 27389942

[32] Singh AA Boyle JR Chetter I , et al. Protamine for heparin reversal after carotid endarterectomy is significantly underutilised: results from a UK national survey. Eur J Vasc Endovasc Surg. 2022;63(4):657–658. doi:10.1016/J.EJVS.2022.01.002. 35184973

[33] Weiler JM Gellhaus MA Carter JG , et al. A prospective study of the risk of an immediate adverse reaction to protamine sulfate during cardiopulmonary bypass surgery. J Allergy Clin Immunol. 1990;85(4):713–719. doi:10.1016/0091-6749(90)90189-B. 2182695

[34] Assadian A Senekowitsch C Assadian O , et al. Antithrombotic strategies in vascular surgery: evidence and practice. Eur J Vasc Endovasc Surg. 2005;29(5):516–521. doi:10.1016/J.EJVS.2005.01.021. 15966091

[35] Zaman SM de Vroos Meiring P Gandhi MR , et al. The pharmacokinetics and UK usage of heparin in vascular intervention. Clin Radiol. 1996;51(2):113–116. doi:10.1016/S0009-9260(96)80267-4. 8631163

[36] Durran AC Watts C. Current trends in heparin use during arterial vascular interventional radiology. Cardiovasc Intervent Radiol. 2012;35(6):1308–1314. doi:10.1007/S00270-011-0337-1. 22245914

[37] Wakefield TW Lindblad B Stanley TJ , et al. Heparin and protamine use in peripheral vascular surgery: a comparison between surgeons of the Society for Vascular Surgery and the European Society for Vascular Surgery. Eur J Vasc Surg. 1994;8(2):193–198. doi:10.1016/S0950-821X(05)80459-1. 8181615

