# Supplementary material for: Perprocedural Heparinization in Non-cardiac Arterial Procedures: The Current Practice in the Netherlands
Source: J Endovasc Ther. 2023 Sep 25;32(4):1009–17. doi: 10.1177/15266028231199714 (PMC12241689; doi:10.1177/15266028231199714)
Supplement: sj-docx-1-jet-10.1177_15266028231199714 – Supplemental material for Perprocedural Heparinization in Non-cardiac Arterial Procedures: The Current Practice in the Netherlands [file sj-docx-1-jet-10.1177_15266028231199714.docx]

Supplemental table 1: ACT devices used per type of procedure.

| **Procedures** | **ACT-protocol** | **Measurement device** |  |  |  |
| --- | --- | --- | --- | --- | --- |
|  |  | *HMS Plus (Medtronic)*  *n (%)* | *ACT Plus (Medtronic)*  *n (%)* | *Hemochron Signature Elite*  *n (%)* | *i-STAT Alinity (Abbot)*  *n (%)* |
| CEA | 8 | 2 (25) | 1 (13) | 5 (53) | - |
| CAS | 1 | - | - | 1 (100) | - |
| EVAR | 10* | 2 (20) | 1 (10) | 7 (70) | - |
| TEVAR | 8 | - | 1 (13) | 7 (88) | - |
| FEVAR/BEVAR | 13 | 1 (8) | 1 (8) | 10 (76) | 1 (8) |
| OR | 10* | 3 (30) | 1 (10) | 6 (60) | - |
| Femoral endarterectomy | 7* | 1 (14) | 1 (14) | 5 (62) | - |
| Femoral-distal bypass | 8* | 1 (13) | 1 (13) | 6 (75) | - |
| Peripheral PTA | 6* | 1 (17) | 1 (17) | 4 (66) | - |

*1 site used APTT monitoring because ACT was not available.

ACT = activated clotting time; CAS = carotid artery stenting; CEA = Carotid endarterectomy; EVAR = endovascular aneurysm repair; FEVAR/BEVAR = fenestrated/branched endovascular aneurysm repair; OR AAA = open abdominal aortic aneurysm repair; PTA = percutaneous transluminal angioplasty; TEVAR = thoracic endovascular aneurysm repair.
